# Transcriptome Profile of the Variegated *Ficus microcarpa* c.v. Milky Stripe Fig Leaf

**DOI:** 10.3390/ijms20061338

**Published:** 2019-03-16

**Authors:** Tin-Han Shih, Szu-Hsien Lin, Meng-Yuan Huang, Wen-Dar Huang, Chi-Ming Yang

**Affiliations:** 1 Biodiversity Research Center, Academia Sinica, Nangang, Taipei 11529, Taiwan; damnos@gate.sinica.edu.tw (T.-H.S.); su043@gate.sinica.edu.tw (S.-H.L.); 2 Department of Horticulture and Biotechnology, Chinese Culture University, Shilin, Taipei 111, Taiwan; HMY6@ulive.pccu.edu.tw; 3 Department of Agronomy, National Taiwan University, Daan, Taipei 101, Taiwan; wendar@ntu.edu.tw

**Keywords:** variegated, *Ficus microcarpa*, transcriptome, *de novo* assembly, RNA-seq, photosynthesis, chlorophyllase

## Abstract

Photosynthetic properties and transcriptomic profiles of green and white sectors of *Ficus microcarpa* (c.v. milky stripe fig) leaves were examined in naturally variegated plants. An anatomic analysis indicated that chloroplasts of the white sectors contained a higher abundance of starch granules and lacked stacked thylakoids. Moreover, no photosynthetic rate was detected in the white sectors. Transcriptome profile and differential expressed gene (DEG) analysis showed that genes encoding PSII core proteins were down-regulated in the white sectors. In genes related to chlorophyll metabolism, no DEGs were identified in the biosynthesis pathway of chlorophyll. However, genes encoding the first step of chlorophyll breakdown were up-regulated. The repression of genes involved in N-assimilation suggests that the white sectors were deprived of N. The mutation in the transcription factor mitochondrial transcription termination factor (mTERF) suggests that it induces colorlessness in leaves of the milky stripe fig.

## 1. Introduction

Leaf variegation is a common feature resulting from the uneven distribution or deficit of photopigments. Variegated leaves are economically important in horticulture and are scientifically significant for plant research. In natural environments, the occurrence of variegation hypothetically enables plants to either adapt to changing light conditions or reduce the possibility of being targeted by herbivores [1]. Variegation might also be a form of mimicry to prevent infestation by insects [2].

Research associated with chlorophyll-deficient variegation has characterized structural, functional, and molecular differences between green and non-green sectors. Abnormal chloroplast morphology and loosened mesophyll arrangements in white (albino) sectors were revealed by studies on the ultrastructure of leaves in variegated figs (*Ficus rubiginosa*) and variegated mutant lines of tobacco (*Nicotiana tabacum*) and *Arabidopsis* (*Arabidopsis thaliana*) [3,4,5]. White sectors also lack photosynthetic activity and show inactivation of fluorescent kinetics and electron transport [6]. Accordingly, white sectors are considered to be heterotrophic (while green sectors are considered to be autotrophic).

Investigations of variegated mutants in *Arabidopsis* and albino mutants in tobacco identified several molecules whose deficits impair the formation of leaf chloroplasts. In the *Arabidopsis* white-green variegated mutant *immutans* (*im*), the lack of terminal oxidase IM in thylakoids leads to irregular organization of lamellar structures, reductions in levels of photopigments, and inhibition of carotenoid formation [7,8,9,10]. Furthermore, FtsHs represent another variegation-related protein family, which normally contributes to progressive protein degradation and maintenance of the thylakoid structure, with plants with loss of FtsHs functions exhibiting distorted plastids and repressed expression of photosynthetic genes in non-green sectors [5,11]. Transcriptome analysis of *im* and *Var2* (an *Arabidopsis* FtsH2 mutant line) demonstrated a downregulation of photosynthesis-related gene expression with elevated levels of oxidative stress-related gene transcripts in the white sectors. A recent transcriptomic study on chlorophyll-deficient mutant leaves of the flamingo flower (*Anthurium andraeanum* Lind.) also showed expressional repression of transcription factors involved in chloroplast development and division [12]. Although those studies of mutant lines improved our understanding of variegation and the effects of chlorophyll deficiency, research on the transcriptional profiles of naturally variegated plants is still lacking.

In the present study, we used milky stripe fig (*Ficus microcarpa*), which has a common variegation with the occurrence of green and white sectors within a single leaf, to examine transcriptomic profiles of naturally variegated leaves. We tested the photosynthesis properties of green and white sectors of leaves and show that the white sectors have negligible responses to light. A transcriptomic assay indicated that the white sectors contain higher abundance of genes related to protein degradation and loss of transcripts for nucleotide and protein synthesis. Differentially expressed gene (DEG) analysis showed that genes encoding the photosystem II (PSII) core proteins were repressed, whereas genes related to chlorophyll degradation were up-regulated. The repression of genes related to N-assimilation and those encoding transcription factors are suggested to be the factors that induce development of white sectors.

## 2. Results and Discussion

### 2.1. Plastid Morphology and Photosynthetic Activity

**Figure 1:**
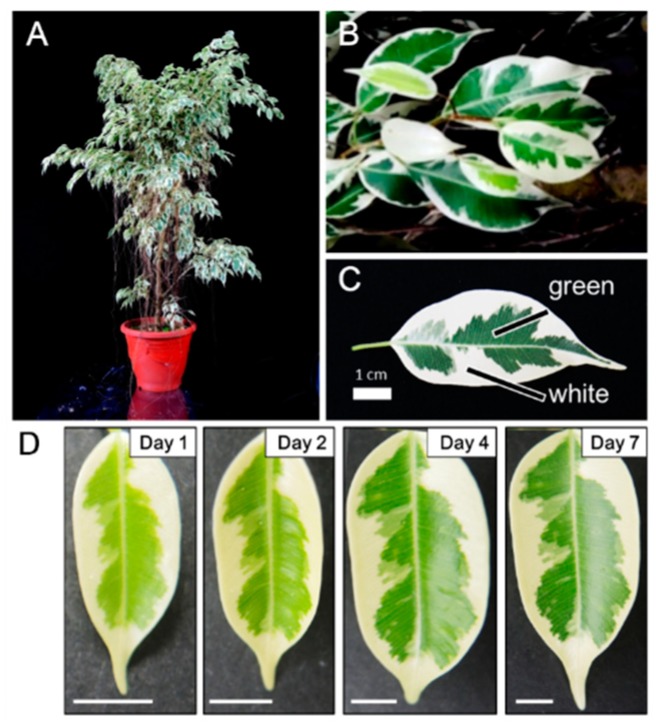
Photographs of (**A**) a whole plant, (**B**) a branch, and (**C**) leaf variegated patterns of the milky stripe fig. Black bars in (**C**) indicate the green and white sectors used in the present study. (**D**) Development of one individual leaf. Scale bar in each panel represented 0.5 cm.

**Figure 2:**
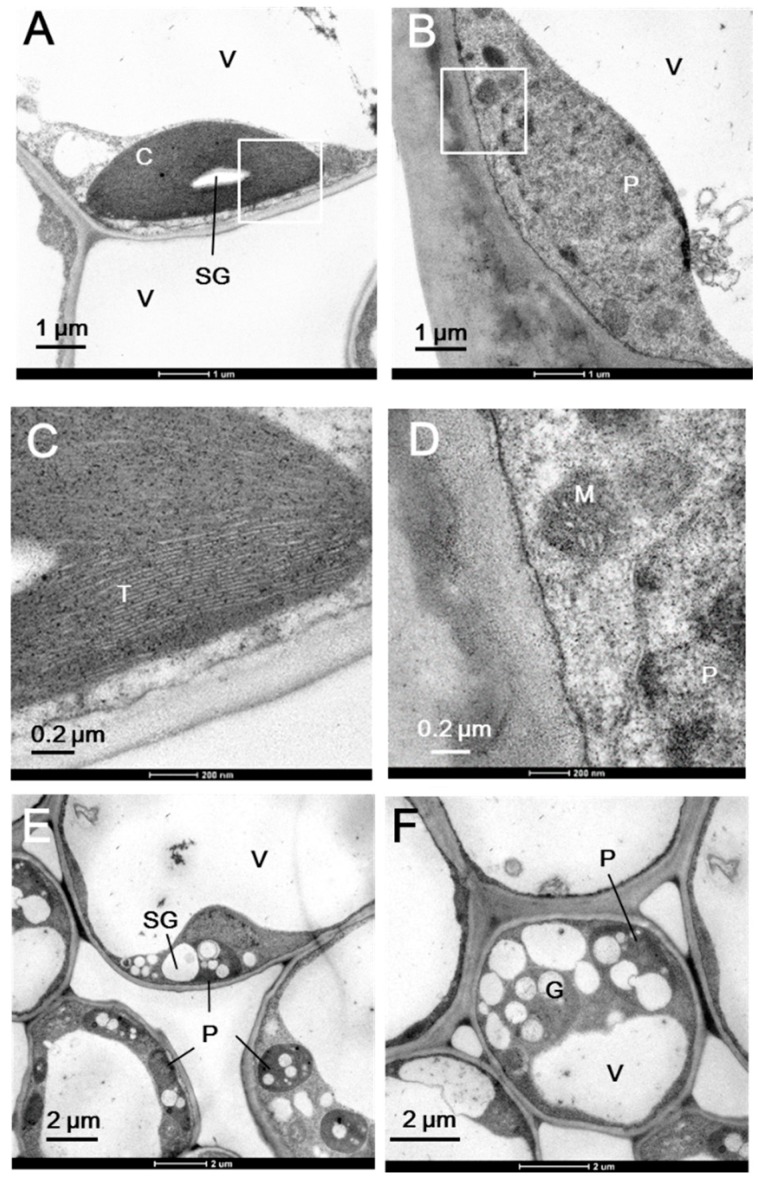
Chloroplast ultrastructure of the green (**A**,**C**) and white (**B**,**D**–**F**) sectors in milky stripe fig leaves. White inserts in (**A**) and (**B**) are shown in (**C**) and (**D**), respectively. C, chloroplast; M, mitochondria; P, plastid; SG, starch granule; T, thylakoid; V, vacuole.

**Figure 3:**
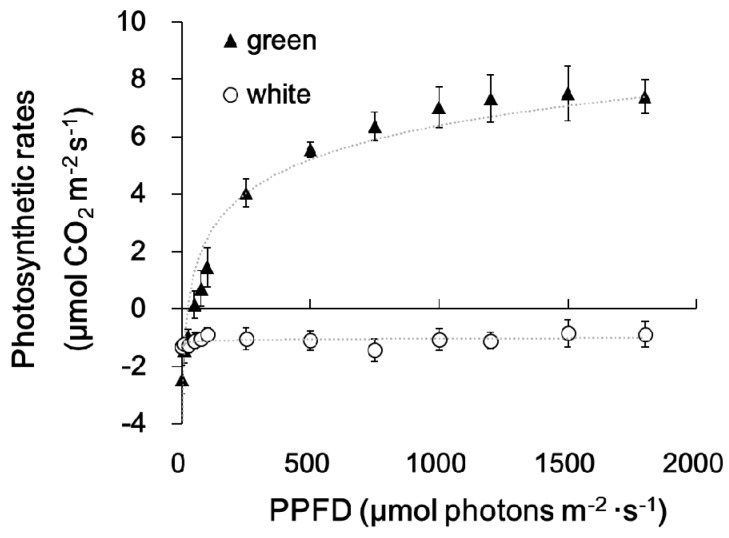
Light response curves of green and white sectors of milky stripe fig leaves. Leaf sectors were treated with light intensity from 0–1800 μmol·m^−2^·s^−1^ photosynthetic photon flux density (PPFD). Data are presented as the mean ± SD (*n* = 3).

Figure 1 shows the morphology of the whole plant (Figure 1A), a branch (Figure 1B), and a single leaf (Figure 1C) of the milky stripe fig. The green-white pattern is fixed through development (Figure 1D). The ultrastructure of plastids in the green and white sectors were analyzed by TEM. Results showed that chloroplasts of the green sectors contained normal stacked grana in the thylakoid (Figure 2A,C). In the white sectors, plastids were found to have rounder shapes with increased abundance of starch granules while lacking stacked grana when compared to that in the green sectors (Figure 2B,D–F). The increment in abundance of starch granules instead of a regular lamellar structure in plastids of the non-green sectors was observed in variegated *Arabidopsis* [3,10,13], tobacco leaves [14], and begonia [15]. The accumulation of starch granules suggests that white sectors are nutrient sinks because they are unable to perform photosynthesis due to the lack of plastids with an organized lamellar apparatus. Deficiency in photosynthetic activities in the white sectors was also found in the milky stripe fig (Figure 3). Responses of the green and white sectors to light were evaluated with light response curves, and the results showed no response in the white sectors according to photosynthetic rates when the ambient light intensity was elevated.

### 2.2. De novo Assembly and Bioinformatic Analysis

In total, 23,679,092 and 22,358,400 reads were obtained for the green (G) and white (W) sectors of leaves, respectively. After de novo assembly and removal of non-coding RNA, 95,394 and 102,861 contigs were assigned to the G and W sectors, respectively (Table 1). The number of contigs was comparable to the ~81,000 contigs assembled in another *Ficus* (*F. carica*; GenBank assembly accession: GCA_002002945.1). After BLAST against the NCBI database, 61,363 contigs were annotated. Finally, 59,602 genes were regarded as expressed genes after trimming off the contigs with zero normalized count values in both green and white sectors (Table 1; for the list of all genes, see Table S1).

**Figure 4:**
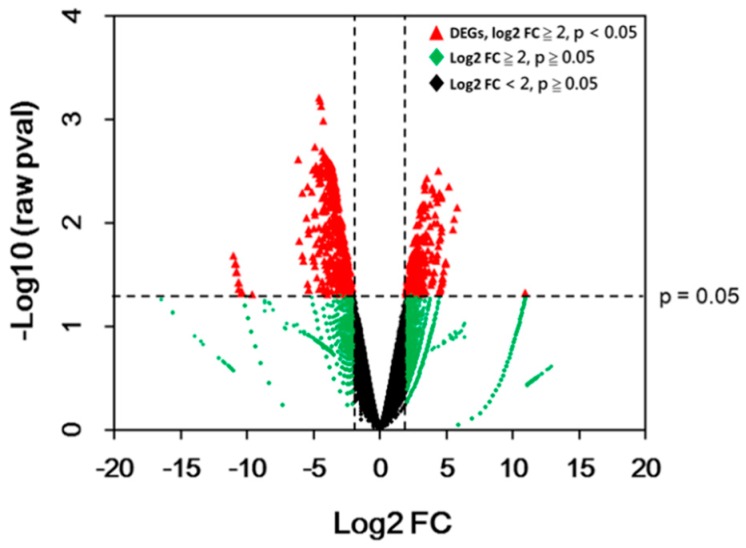
Volcano plot of genes in white versus green sectors. Data for all genes are plotted as log2 fold change (FC) versus the –log10 of raw *p*-value (pval). Genes that were considered differential expressed genes (DEGs) are highlighted as a red dot. The horizontal dashed line indicates the significant threshold of the *p*-value, whereas the vertical dashed lines represent the threshold of log2 fold change = 2 or −2.

General gene expressions are depicted in a volcano plot (Figure 4). The analysis of differential expression was performed on all genes through DESeq2 Bioconductor packages using a threshold Log2 fold change of 2 (absolute fold change 4) with raw *p*-value < 0.05. A total of 467 DEGs were obtained after the analysis. Among them, 183 genes were up-regulated, whereas 284 genes were down-regulated (Table 1; for the list of DEGs, see Table S2). This result is similar to that for the white sector of the Arabidopsis *var2* mutant, which was found to have a higher number of downregulated genes compared to the green sector [16].

### 2.3. Differential Expressed Genes (DEGs)

#### 2.3.1. Photosynthesis and Chlorophyll Metabolism

**Figure 5:**
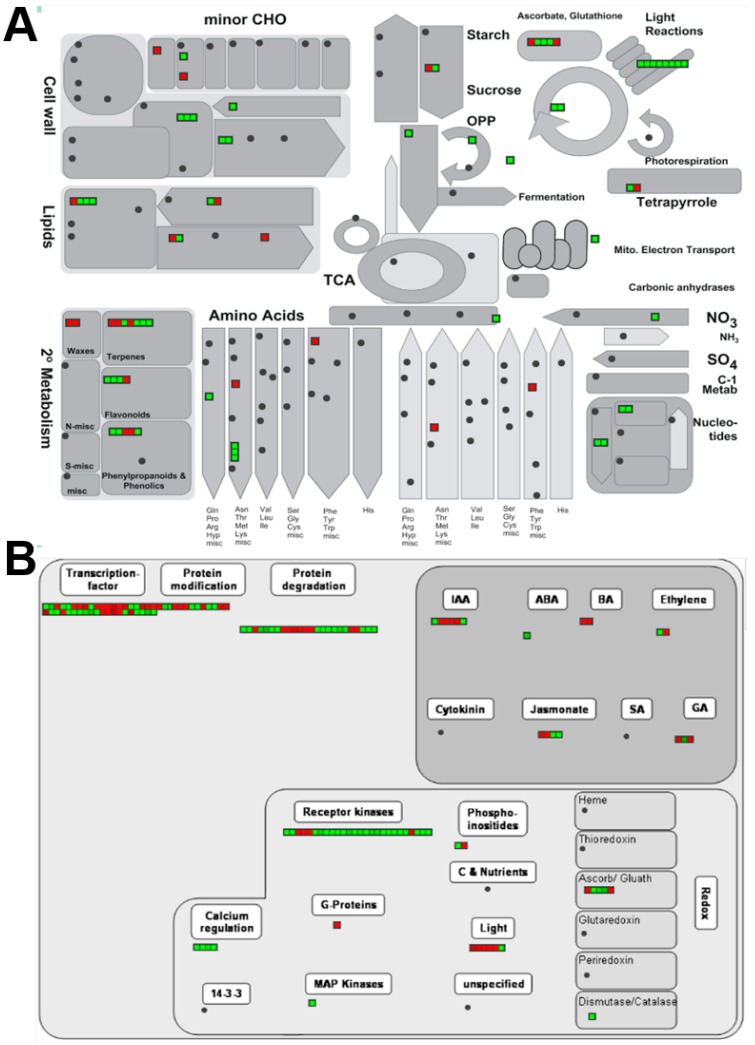
MapMan overview of (**A**) metabolic- and (**B**) regulatory-related DEGs. Each bin represents a single DEG. Red and green indicate up- or down-regulation, respectively.

The DEGs in different metabolic and regulatory pathways were visualized in MapMan (Figure 5) by applying the corresponding *Arabidopsis* orthologues. Results showed that DEGs related to photosynthesis (including light reaction and Calvin cycle) were all down-regulated. Genes that encode PSII core proteins, including *psba*, *psbb*, *psbc*, and *psbd*, were all down-regulated (Table 2). The transcript encoding the RuBisCO large subunit displayed decreased abundance as well. The expressional pattern of photosynthesis-associated genes in the present study was slightly different from the array-based analysis of *Arabidopsis immutans*, which showed that at least one-third of PS genes were down-regulated in white sectors. Furthermore, the nuclear-encoded cytochrome c6 was increased in *Arabidopsis immuntans* [17], but the gene of the plastid-encoded cytochrome c6 subunit in the present study was down-regulated. Notably, the photosynthesis-associated DEGs are all plastid coded.

In white sectors of the milky stripe fig, the genes that encode enzymes that participate in chlorophyll biosynthesis showed no differential expressions. In studies of albino mutants of *Arabidopsis*, repression of transcript content was found in genes related to enzymes that regulated key steps of the chlorophyll synthesis [17,18]. Transcriptome analysis of *A. andraeanum* revealed that two-thirds of chlorophyll synthesis-related DEGs in mutant non-green leaves were also down-regulated [12]. Our data indicate that the absence of green color in milky stripe fig might be caused by other factors.

In white sectors of milky stripe fig leaves, genes related to enzymes that mediate critical processes of chlorophyll degradation were found to be differentially expressed (Table 2). Expression of *clh1* and *clh2*, that encode chlorophyllase I and II, respectively, which initiate degradation by removing phytol from chlorophyll, were remarkably increased. Both chlorophyllase I and II were found to be able to use chlorophyll a and b as substrates [19]. These results were in line with our previous findings indicating that chlorophyll a and b degradation rates were higher in white sectors of milky stripe fig leaves [20]. Our results suggest that the loss of chlorophyll in leaves might not only be in pace with the deficit in chlorophyll biosynthesis but might be induced by reinforced chlorophyll degradation.

An alternative step for starting chlorophyll breakdown is to remove magnesium from heme in order to form pheophytin. This dechelating process is facilitated by the enzyme stay-green (SGR) [21,22]. In white sectors of the milky stripe fig, the SGR-coding genes, *non-yellowing 1/stay-green 1* (*nye1*/*sgr1*) and *stay-green like* (*sgrl*), were up-regulated 8.8- and 13-fold, respectively (Table 2). Previous reports have demonstrated the divergent function of SGR1 and SGRL in the regulation of growth and aging [23]. The transcript amount of SGRL was higher in developing leaves, whereas the expression of SGR1 was increased in senescence leaves. The up-regulation of *sgr1* and *sgrl*, together with the elevated expression of *clh1* and *clh2*, indicates that an urgent breakdown of chlorophyll is required. In addition, *nyc1,* which encodes chlorophyll b reductase (CBR) that degrades chlorophyll b, was also up-regulated 5.4-fold. This might be caused by the increase of SGR, whose overexpression was recently suggested to activate the expression of *nyc1* that facilitates the conversion of chlorophyll b to chlorophyll a [24]. Reduced chlorophyll content might affect photosystem functions because chlorophylls are able to directly accumulate PSII D1 and CP43 proteins by enhancing the stability of the chlorophyll apoprotein [25]. In the present study, however, we suggest that increased Chl degradation might be a consequence of the lack of PSII core protein. A PSII deficiency could lead to an elevation of free chlorophyll abundance, which is recognized as redundant chlorophyll that is subsequently degraded by CBR, chlorophyllase, or SGR.

#### 2.3.2. Chloroplast Organization

Despite the deficit in the photosystem, the malfunction of chloroplast organization might be another key factor that induced the whiteness in the leaves of the milky stripe fig. Previous research has already identified the transcription factors which, in mutants, lead to albino phenotypes in leaves [7,26]. However, no repression of these factors was identified in the present study. Instead, a group of mitochondrial transcription termination factor (mTERF) genes were remarkably down-regulated. These mTERFs were suggested to play a crucial role in the determination of the development of white sectors (Table 2). The characterization of plant’s mTERFs was initiated in recent years and has demonstrated the regulatory significance of mTERFs in chloroplasts [27,28,29]. Moreover, mTERF mutants altered the development of chloroplasts and the morphology leaves. The paleness of leaves in mTERF mutants was also demonstrated [30]. Therefore, it is likely that the repression of mTEFRs in the leaves of the milky stripe fig resulted in the observed whiteness.

#### 2.3.3. Nitrate Metabolism

Nitrate is the most abundant inorganic molecule for acquiring nitrogen in plant tissues. The uptake of nitrate relies on the nitrate transporter (NRT), while the incorporation of nitrate to form NH_4_^+^ is regulated by nitrate reductase (NR) and nitrite reductase (NiR), which are involved in the first and second steps of the nitrate assimilation pathway, respectively [31]. In this study, DEG analysis revealed that the expression of *nrt1* and *nir1* was repressed in white sectors. Although genes encoding NR were not found in the DEG analysis, the transcript amount of NR- and NR2-coding genes in white sectors also showed a decrease of more than 300-fold (Table S1, Fm_contig_54471 and Fm_contig_1786; DESeq2 *p*-value = 0.0507 and 0.0599, respectively). Decreases of NRT, NR, and NiR might result in a limitation of nitrogen, and thus induce chlorosis and inhibit chloroplast protein translation [32]. In *Arabidopsis* albino leaves, expressions of *NR* and other nitrogen metabolism-associated genes were increased, accompanied by the promotion of nitrate assimilation and NH_4_^+^ production [18]. Structural analysis of the *NR* mutant of *Nicotiana plumbaginifolia* showed that the starch content of plastids was elevated [33], similar to the findings of the present study. Therefore, it is possible that the occurrence of white sectors in milky stripe fig leaves is induced by a NR deficiency. Further physiological analyses are needed to confirm if NR and NiR induce whiteness or are only correlated with it.

The uroporphyrinogen III methyltransferase (UPM1)-coding *upm1* is another N assimilation-related gene that was repressed in white sectors (Table 2). Uroporphyrinogen III methyltransferase catalyzes the rate limiting step of the biosynthesis of siroheme, which was found to regulate the assimilation of nitrogen and sulfite in plants [34]. In *Arabidopsis*, knockout of *UPM1* induced lethal effects, whereas its overexpression increased total protein content and enhanced photosynthesis [35]. Transcription of *UPM1* was able to be induced by nitrate [36]. In a similar fashion, decreased *nir1*, *nrt1*, and *upm1* (as well as *nr1* and *nr2*) suggests that the N-assimilation function in white sectors was deprived.

**Figure 6:**
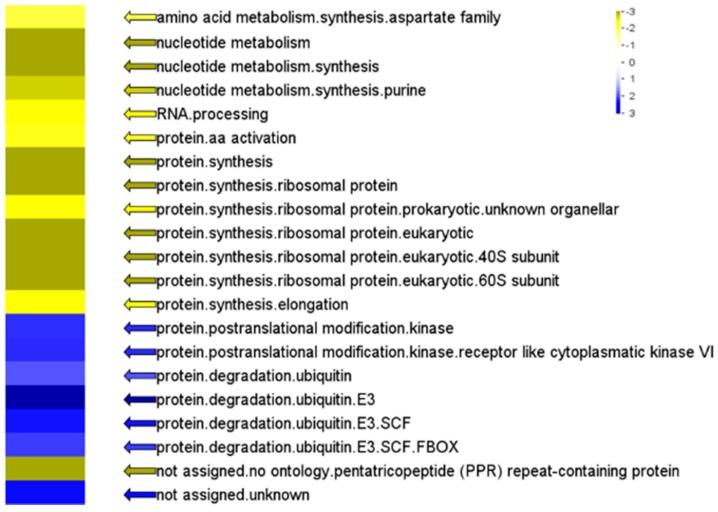
Over-represented analysis (ORA) of genes of the variegated milky stripe fig leaf. Functional gene categories (and sub-categories) of green and white sectors were determined by PageMan. Blue and yellow represent up- and down-regulated genes which are significantly overrepresented, respectively. Values were false color coded using a scale of −3 to +3.

However, the downregulation of *NR* and *NiR* in white sectors might be the result of higher protein degradation rates (Figure 6). Protein degradation was suggested to induce the production of ethylene, a hormone that gives rise to the accumulation of NH_4_^+^ in cell fluids [37,38]. Thus, elevated NH_4_^+^ induces negative feedback on *NR* and *NiR* expressions. In addition, an induced protein degradation rate might lead to the accumulation of glutamine, which results in the repression of the expression of *NR* [39,40]. These conjectures are supported by observations showing that in albino leaves of *Arabidopsis* mutants, NH_4_^+^ and glutamine contents are higher than those in green leaves [18].

### 2.4. Over-Represented Analysis (ORA)

The ORA of preference gene expressions in different functional categories showed that the expression of genes involved in cellular metabolism were massively changed in white sectors (Figure 6). Genes related to nucleotide and amino acid metabolism, as well as protein synthesis, were repressed. Additionally, the expression of genes related to protein degradation was found to be elevated, exhibiting a preference for protein ubiquitination functions. We suggest that the observed pattern of silenced protein synthesis and active protein degradation represents the machinery that cells in white sectors use to deal with the micro- and macro-molecules related to photosynthesis. To our knowledge, no transcriptomic analysis of albino leaf sectors has revealed such tremendous changes in genes related to fundamental cell metabolism. This result, comprising chlorophyll breakdown, deficit photosynthesis, and degradation of proteins and nucleic acids of white sectors indicates a senescence phenomenon. Either a cause or a result, the senescence-related factor present in white sectors needs further examination.

The increased expression of genes related to ubiquitination might also be a mechanism to eliminate damaged proteins that were attacked by reactive oxygen species (ROS). Accumulation of ROS in the leaves of green-white *var2* mutants was previously reported [41]. As amino acids are attacked by ROS, oxidized proteins are susceptible to ubiquitination and, subsequently, to degradation [25,26]. Furthermore, E3 proteins were also shown to act as the main enzymes mediating tolerance to ROS-inducing abiotic stress by the regulation of the expressions of downstream transcription factors [27,28].

### 2.5. Real Time Quantitative PCR

**Figure 7:**
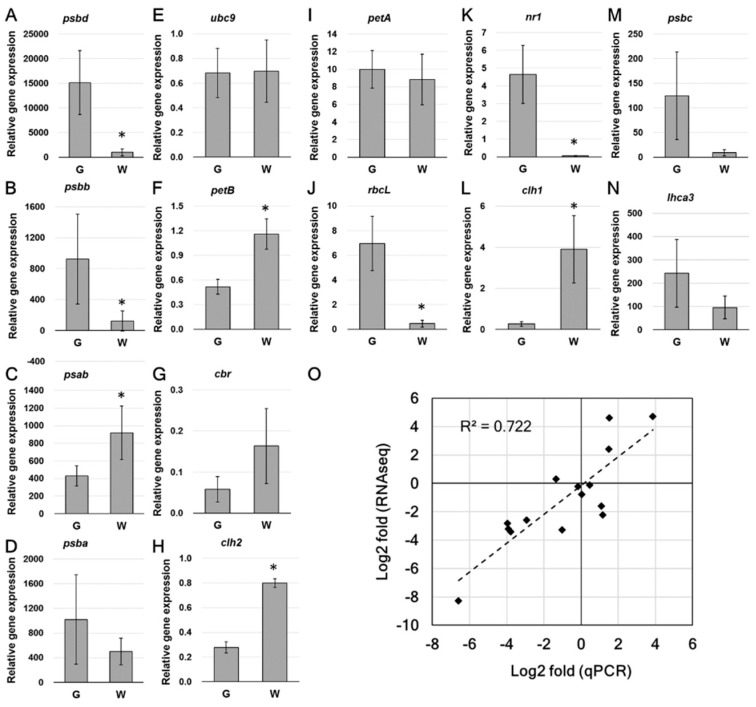
Real-time qPCR and the relationship of qPCR and RNAseq fold change. (**A**–**N**), qPCR analysis of the relative expression of selected DEGs. (O), Relationship of qPCR and RNAseq fold change. Folds are presented as Log2 value. Data are presented as the mean ± SD (*n* = 5–6). * Significant difference between green and white sectors (*t*-test, *p* < 0.05).

The expression of several interesting genes was subjected to qPCR to verify the transcript level (Figure 7). Real time qPCR analysis showed that the expression of PSII-related DEGs, including *psbd* and *psbb* (Figure 7A,B), were significantly decreased in white sectors. Although no statistical difference was found in the expression of *psbc*, the average transcript amount was reduced in white sectors (Figure 7M). The PSI core subunit-coding *psab*, which was not recognized as a DEG in RNAseq analysis, showed a significant up-regulation (Figure 7C). The expressional pattern of other interesting genes, including *rbcL*, *nr1*, *clh1*, and *clh2* was consistent with RNAseq data. The relationship of multiple changes obtained from the qPCR and RNA-Seq (normalized by DESeq2) analyses is plotted in Figure 7O. Log2 multiples of changes are shown in Table S3.

## 3. Material and Methods

### 3.1. Plant Material

Milky stripe fig (*Ficus microcarpa* cv. milky strip) plants were purchased from a local nursery farm and grown in a greenhouse with natural light for 1 month. The average temperature during sampling months was 28.6 °C. Only sunny and mature variegated leaves were used for the experiments. Plant morphology and leaf sectoring are shown in Figure 1.

### 3.2. Light Response Curve

Light response curves of photosynthetic activity were measured with a portable, open-flow gas exchange system connected to a leaf chamber and an LED light source (model 6400XT, LI-COR, Lincoln, NE, USA) with a small leaves chamber (6400-40 chamber, area = 2 cm^2^). Leaf sectors were subjected to light from low to high levels of PPFD (2, 10, 25, 50, 75, 100, 250, 500, 750, 1000, 1200, 1500, and 1800 μmol/m^2^/s) and CO_2_ concentration was maintained at 400 µmol·mol^−1^. Data were obtained when the exchange of CO_2_ was stable (about 5 min under each level of illumination). Natural logarithms were used to fit the light response curves. All measurements were taken before 11:00 a.m. to avoid the midday depression in photosynthesis.

### 3.3. Transmission Electron Microscopy (TEM)

Green and white sectors of leaves were cut into small cubes in the field and placed in a fixation solution containing 2.5% glutaraldehyde and 4% paraformaldehyde in 0.1 M sodium phosphate buffer (pH 7.0). Samples underwent 20 min of rinsing three times and were post-fixed in 1% osmium tetroxide for 2 h. After being dehydrated through an ethanol series, samples were infiltrated and embedded in Spurr’s resin [42] and then polymerized at 70 °C for 8 h. Ultrathin sections (~70–90 nm) were collected and stained with ethanol uranyl acetate and lead citrate. The morphology of plastids was observed with a Philips CM 100 TEM (Amsterdam, The Netherlands) at 75 kV.

### 3.4. RNA Purification, Complementary cDNA Library Construction, and Transcriptome Sequencing

The total RNA of green (G) and white (W) sectors was prepared from a single leaf using an E.Z.N.A. Plant RNA Kit (Omega Bio-tek, Norcross, GA, USA), according to the manufacturer’s protocol. Three paired-end cDNA libraries for the G and W sectors were constructed for transcriptome sequencing. cDNA libraries were sequenced on an Illumina MiSeq platform (Illumina, San Diego, CA, USA). Ambiguous nucleotides, adapter sequences, and low-quality sequences were trimmed from the RAW reads. cDNA library construction and transcriptome sequencing were performed by a commercial service provider (Tri-I Biotech, New Taipei City, Taiwan).

### 3.5. De novo Assembly, BLAST, and RNA-Sequencing (RNA-Seq)

Paired-end reads were assembled and analyzed in CLC Genomics Workbench v. 7.5 (CLC bio, Aarhus, Denmark, now QIAGEN). Non-coding RNA contigs were removed based on a BLAST search of contigs against a reference (Rfam). Pair-end sequencing results were deposited in NCBI’s Sequence Read Archive (reference: SRP131635). An *Arabidopsis* database (The Arabidopsis Information Resource, TAIR) was also searched for specific annotations of expressed genes. Genes with the highest bitscore were kept when multiple *F*. *microcarpa* genes were mapped onto duplicate *Arabidopsis* orthologs. The DESeq2 Bioconductor package was applied to determine the differential expressed gene (DEG) [43]. An analysis of the preference of functional gene categories was performed with MapMan (v. 3.5.1, with PageMan integrated [44]) by imputing the ID of *Arabidopsis orthologues*. The Wilcoxon statistical test was applied with the Benjamini–Hochberg procedure.

### 3.6. Quantitative Reverse-Transcription Polymerase Chain Reaction (RT-qPCR)

One microgram of total RNA extracted from each sector was used for cDNA synthesis. cDNA synthesis was performed using a Transcriptor First Strand cDNA Synthesis Kit (Roche, Basel, Switzerland) with oligo(dT) and random hexamers as primers. Primer sets for the target and reference genes are listed in Table S4. The qPCR was performed with the StepOne Plus Real-Time PCR system (Thermo Fisher Scientific, Waltham, MA, USA) with Roche FastStar Universal SYBR Green Master reagent (Roche). Relative gene expression values are presented as 2^−△*C*t^, with △*C*t calculated by subtracting the target gene *C*t from the *C*t of reference gene *more axillary branches 2* (*max2*). The multiple of change of each gene in leaf tissues was calculated by 2^−△*C*t^_W_/2^−△*C*t^_G_.

## 4. Conclusions

Transcriptome profiles in white sectors of variegated milky stripe fig leaves have several patterns distinct from those found in the variegated *Arabidopsis* mutant lines. The increased expression of genes related to chlorophyll breakdown in albino sectors of leaves represents a novel finding. We suggest that the deficit of transcription factor mTERF might be a key factor that induces variegation. The depression of genes encoding N-assimilation-related proteins might contribute to the deprivation of nitrogen and result in colorless. As expressional preference of genes related to ubiquitination was found in white sectors, the lack of photosynthesis-related proteins might be explained by higher degradation rates. The increase in ubiquitin-associated protein catabolism in albino sectors of *Arabidopsis* mutants further supports our suggestion [45]. However, determining whether photosynthetic proteins are highly ubiquitinated in white sectors still requires further investigation.

## Figures and Tables

**Table 1:** Results of Next Generation Sequencing (NGS), RNAseq and DEG analysis in green (G) and white (W) sectors.

NGS and de novo Assembly	
Reads of G	23,679,092
Reads of W	22,358,400
**De novo assembled contig**	
G	95,394
W	102,861
**RNASeq**	
Annotated contigs	61,363
Genes after trim	59,602
DEGs	467
up-regulated DEGs	183
down-regulated DEGs	284

**Table 2:** List of DEGs related to photosynthesis and N-assimilation.

Symbol	Log 2 Fold Change	At Orthologue	Description
Photosystem			
*psba*	−3.3	ATCG00020	PSII D1 protein
*psbb*	−2.6	ATCG00680	CP47 subunit of the PSII
*psbc*	−3.4	ATCG00280	CP43 subunit of the PSII
*psbd*	−3.2	ATCG00270	PSII D2 protein
*psbh*	−2.4	ATCG00710	PSII reaction center protein H
*psaa*	−2.3	ATCG00350	PsaA protein comprising the reaction center for PSI
*rbcl*	−2.8	ATCG00490	Large subunit of RUBISCO
*petb*	−2.2	ATCG00720	Cytochrome b(6) subunit of the cytochrome b6f complex
*ndhb.2*	−3.0	ATCG01250	NADH dehydrogenase ND2
Tetrapyrrole metabolism			
*upm1*	−3.2	AT5G40850	Urophorphyrin III methylase
*nyc1*	2.4	AT4G13250	Chlorophyll b reductase
*clh1*	4.7	AT1G19670	Chlorophyllase I
*clh2*	4.6	AT5G43860	Chlorophyllase II
*nye1*	3.1	AT4G22920	Non-yellowing 1, stay-green 1
*sgrl*	3.7	AT1G44000	Stay-green like protein
Chloroplast organization			
*hcf173*	3.4	AT1G16720	High chlorophyll fluorescence phenotype 173
*sig5*	2.8	AT5G24120	Sigma factor 5
*sco3*	−3.1	AT3G19570	Snowy cotyledon 3, QWRF domain containing 1
*mterf9*	−10.4	AT5G55580	Mitochondrial transcription termination factor (mTERF) 9
*emb2219*	−3.2	AT2G21710	Embryo defective 2219 (mTERF)
mterf*	−2.4	AT1G78930	Mitochondrial transcription termination factor (mTERF)
Nitrate metabolism			
*nir1*	−2.9	AT2G15620	Nitrite reductase 1 (NiR1)
*nrt1*	−2.4	AT1G69850	Nitrate transporter 1 (NRT1)
*xip1*	−4.1	AT5G49660	Xylem intermixed with phloem 1

* no specific name assigned.

## Supplementary Materials

The following are available online at https://www.mdpi.com/1422-0067/20/6/1338/s1.
